# Early Ovarian Reserve Depletion During Neoadjuvant Chemotherapy in Female Patients with Bone and Soft Tissue Sarcoma: A Longitudinal Anti-Müllerian Hormone Study

**DOI:** 10.3390/cancers18111821

**Published:** 2026-06-02

**Authors:** Boyang Wang, Huimin Liu, Xiangyu Liu, Shidong Wang, Wei Guo, Jichuan Wang, Xin Sun

**Affiliations:** 1 Musculoskeletal Tumor Center, Beijing Key Laboratory for Musculoskeletal Tumors, Peking University People’s Hospital, Beijing 100044, China; 2 Multidisciplinary Diagnosis and Treatment Center for Bone Tumors, Peking University Shougang Hospital, Beijing 100144, China; 3 Body Sculpture and Fat Transplantation Center, Plastic Surgery Hospital (Institute), Peking Union Medical College, Chinese Academy of Medical Sciences, Beijing 100144, China

**Keywords:** bone and soft tissue sarcoma, anti-Müllerian hormone, ovarian reserve, neoadjuvant chemotherapy, fertility preservation, gonadotoxicity

## Abstract

**Background/Objectives**: Bone and soft tissue sarcomas frequently affect children, adolescents, and young adults, yet data on chemotherapy-related ovarian reserve decline in this population remain limited. We aimed to characterize anti-Müllerian hormone (AMH) dynamics during treatment and identify factors associated with post-neoadjuvant chemotherapy (post-NACT) ovarian reserve. **Methods**: We conducted a retrospective, single-center, longitudinal cohort study of 85 female patients with bone and soft tissue sarcoma who underwent serial serum AMH testing during treatment. In the paired cohort with complete baseline and post-NACT AMH measurements (*n* = 75), post-NACT AMH was defined as the value obtained at the surgery/post-neoadjuvant time point after completion of NACT. Log-transformed multivariable linear modeling and left-censored Tobit regression sensitivity analysis were performed. **Results**: Median AMH declined from 3.46 ng/mL at baseline to 0.39 ng/mL after the first NACT cycle and to 0.15 ng/mL after the second cycle, corresponding to reductions of 88.7% and 95.7%, respectively. In the paired cohort, median AMH declined from 3.81 ng/mL to 0.19 ng/mL post NACT, with a median absolute decline of 2.75 ng/mL and a median percent decline of 94.4%. In the log-transformed multivariable linear model, log(baseline AMH) was the sole independent predictor of log(post-NACT AMH) (β = 0.735, 95% CI 0.357–1.114, *p* < 0.001). In Tobit sensitivity analysis, 4 post-NACT AMH values were treated as left-censored at 0.05 ng/mL, and the pattern remained consistent. **Conclusions**: Ovarian reserve depletion in female sarcoma patients occurred predominantly within the first one to two cycles of NACT. These findings support early, individualized fertility preservation counseling in this population.

## Simple Summary

Bone and soft tissue sarcomas often affect children, adolescents, and young adults, but the impact of chemotherapy on ovarian reserve in this population remains poorly defined. In this real-world longitudinal study, we monitored serum anti-Müllerian hormone (AMH), a sensitive marker of ovarian reserve, in 85 female patients with sarcoma during treatment. AMH declined sharply within the first one to two cycles of neoadjuvant chemotherapy, indicating that ovarian reserve damage occurs very early. In the paired cohort, AMH remained markedly suppressed at the completion of neoadjuvant chemotherapy. Baseline AMH was the sole independent predictor of post-treatment ovarian reserve in the fully adjusted log-transformed multivariable model; no treatment-related covariate reached statistical significance. These findings highlight the narrow window for fertility preservation and support early, individualized counseling according to pubertal status and treatment urgency.

## 1. Introduction

Bone and soft tissue sarcomas—including osteosarcoma, Ewing sarcoma, and rhabdomyosarcoma—collectively represent approximately 11% of all cancers diagnosed in adolescents and young adults (AYA) aged 15–29 years and 6–15% of all pediatric malignancies, constituting the second most common category of solid tumors in the pediatric and AYA population, with peak incidence between 10 and 25 years of age [1,2,3]. Modern multiagent chemotherapy regimens incorporating alkylating agents (cyclophosphamide [CTX], ifosfamide [IFO]), anthracyclines (doxorubicin [DOX]), and platinum compounds (cisplatin [DDP]) have substantially improved long-term survival, yet these agents carry well-documented gonadotoxic potential. In the largest pediatric cancer survivorship cohort to date, Chemaitilly et al. [4] reported an overall premature ovarian insufficiency (POI) prevalence of 10.9% among 921 female childhood cancer survivors, with high-dose alkylating agent exposure identified as the strongest independent risk factor. In a European multicenter study of Hodgkin lymphoma survivors, van der Kaaij et al. [5] demonstrated that the cumulative POI risk reached 60% in patients receiving alkylating agent-containing regimens, compared with only 3% in those treated with non-alkylating protocols. Thus, the preservation of reproductive potential in young women with bone and soft tissue sarcomas must be integrated into treatment planning from the point of diagnosis.

Against this clinical background, anti-Müllerian hormone (AMH) has emerged as the most sensitive serum marker for quantifying chemotherapy-induced ovarian reserve depletion, owing to its cycle-independent stability and direct reflection of the primordial and small antral follicle pool [6]. Prospective longitudinal studies in breast cancer and lymphoma cohorts have consistently demonstrated that AMH levels decline precipitously within the first chemotherapy cycles, often falling to undetectable concentrations, with subsequent recovery trajectories that vary markedly by regimen intensity and patient age [7,8]. Anderson et al. [9] in a secondary analysis of the RATHL trial, confirmed that age is a critical determinant of post-treatment ovarian function recovery, with patients older than 35 years exhibiting significantly diminished recovery capacity. However, a fundamental challenge persists: bone and soft tissue sarcoma-specific chemotherapy regimens frequently combine multiple high-gonadotoxicity agents at cumulative doses exceeding conventional thresholds (CTX equivalent dose > 8000 mg/m^2^), yet virtually no prospective data exist on the temporal dynamics of ovarian reserve depletion and recovery in this population.

Several converging lines of evidence delineate the current knowledge landscape—and its critical boundaries. Mechanistic studies have established that alkylating agents and platinum compounds induce direct follicular apoptosis, granulosa cell injury, and stromal fibrosis, leading to irreversible acceleration of follicle pool depletion [10]. In the clinical domain, Decanter et al. [8] conducted the most comprehensive longitudinal AMH study in young (15–35 years old) lymphoma patients to date (*n* = 122), demonstrating profoundly delayed recovery in patients receiving alkylating agent-intensive regimens such as BEACOPP, while patients on ABVD exhibited near-complete AMH restoration within 12 months. These findings corroborate earlier observations by Dezellus et al. [7], who reported that only 45% of 250 breast cancer patients who were significantly younger and had significantly higher basal AMH levels showed partial AMH recovery at 24 months. On the protective intervention front, meta-analytic evidence supports Gonadotropin-releasing hormone (GnRH) agonist co-administration as a strategy to reduce POI risk [11,12], though its effect on downstream fertility endpoints—spontaneous pregnancy rate and live birth rate—remains inconclusive. Crucially, these data derive almost exclusively from breast cancer and lymphoma populations. The degree to which drug-specific gonadotoxicity profiles, dose–response relationships, and recovery trajectories can be extrapolated to bone and soft tissue sarcomas patients—who receive distinct multi-agent combinations (MAP: methotrexate [MTX], anthracyclines plus platinum compounds, or VDC/IE: vincristine, DOX and CTX followed by ifosfamide [IFO] and etoposide [VP-16]) at disease-specific cumulative doses and at characteristically younger ages—remains entirely unresolved.

In the present study, we conducted a real-world, longitudinal observation of AMH dynamics during chemotherapy in 85 female patients with bone and soft tissue sarcoma. In the subset of patients with paired AMH measurements, we further analyzed risk factors associated with post-NACT AMH during neoadjuvant chemotherapy. To our knowledge, this study provides the sarcoma-specific, longitudinally characterized dataset defining the kinetics and determinants of chemotherapy-induced ovarian reserve depletion, offering an evidence base for risk-stratified fertility preservation counseling in this vulnerable population.

## 2. Materials and Methods

### 2.1. Patient Population

This was a retrospective, single-center, longitudinal cohort study designed to characterize the dynamic changes in ovarian reserve during and after chemotherapy in female patients with bone and soft tissue sarcoma. Between August 2020 and March 2025, a total of 92 female patients with histologically confirmed bone or soft tissue sarcoma who underwent serum AMH testing were identified at Peking University People’s Hospital. Of these, 4 patients were excluded for recurrent disease at the time of enrollment and 3 were excluded for incomplete treatment courses, yielding a final cohort of 85 patients. The cohort comprised 65 osteosarcoma patients (76.5%), 10 Ewing sarcoma patients (11.8%), and 10 patients with other sarcoma subtypes (11.8%). The patient selection process is summarized in Supplementary Figure S1. Of the 85 eligible patients, a paired cohort was defined as those with complete AMH measurements at both the baseline (pre-chemotherapy) and post-NACT (surgery/post-neoadjuvant) time points from the same individual (*n* = 75). This paired design allowed within-patient quantification of ovarian reserve decline across the neoadjuvant treatment course and served as the analytic cohort for multivariable modeling.

### 2.2. Treatment

Treatment decisions were made by a multidisciplinary sarcoma team in accordance with institutional protocols and contemporary guidelines. NACT regimens were determined by histological diagnosis: osteosarcoma patients predominantly received the MAP regimen (high-dose MTX [HD-MTX], DOX, and DDP) or MAP-IE (MAP plus IFO and VP-16); Ewing sarcoma patients received the VDC/IE regimen (vincristine, DOX, and CTX alternating with IFO and VP-16); other sarcoma subtypes were treated according to histology-specific protocols. Following NACT, surgical resection was performed when indicated, with the approach (limb-sparing with or without reconstruction, or amputation) determined by tumor extent and response to NACT. ACT was administered post-operatively according to standard protocols.

For each patient, cumulative doses of the following chemotherapeutic agents were recorded separately for the NACT and ACT phases: CTX, IFO, platinum agents (cisplatin or carboplatin), anthracyclines (doxorubicin or epirubicin, expressed as DOX equivalents), MTX, VP-16, and vinca alkaloids (vincristine). All cumulative doses were normalized to body surface area (BSA; mg/m^2^) to enable inter-patient comparison and dose–response analysis.

The cumulative gonadotoxic burden was quantified using the Alkylating Agent Dose (AAD) score, a validated composite scoring system that integrates cumulative exposures to alkylating agents and other gonadotoxic compounds into a single ordinal metric [13]. The AAD score has been widely applied in pediatric and young adult oncology research to stratify the risk of POI following chemotherapy. Patients were classified as high gonadotoxic exposure (AAD ≥ 3) or low-to-moderate exposure (AAD < 3). The AAD score was calculated for each patient based on their complete treatment course (NACT plus ACT).

### 2.3. AMH Measurement

Serum AMH was selected as the primary endpoint for ovarian reserve assessment based on its established superiority over FSH and estradiol as a cycle-independent, sensitive marker of the primordial and small antral follicle pool. Peripheral venous blood samples were collected at the following pre-specified time points: (i) baseline, defined as pre-chemotherapy (prior to the first NACT cycle); (ii) serial time points during NACT; and (iii) during ACT, including post-ACT cycle 6 and subsequent assessments as clinically available. Blood samples were collected, allowed to clot at room temperature, and centrifuged. Serum was aliquoted and stored at −80 °C until batch analysis. AMH concentrations were measured using a validated electrochemiluminescence immunoassay with a lower limit of detection of 0.05 ng/mL. To provide normative context for the interpretation of observed AMH values, age-stratified AMH reference intervals from healthy population studies were referenced. For females aged 6–9 years, the reference median AMH is approximately 3.2 ng/mL; for those aged 9–12 years, approximately 4.2 ng/mL; and for adolescents and young adults aged 15–35 years, values typically range from 2.5 to 5.0 ng/mL [14,15,16]. All measurements were performed in a single accredited laboratory to minimize inter-assay variability. Values below the assay detection limit were assigned the detection-limit value (0.05 ng/mL) for descriptive summaries and statistical analyses. Post-NACT AMH was defined as the AMH measurement obtained at the surgery/post-neoadjuvant time point after completion of neoadjuvant chemotherapy.

### 2.4. Data Collection and Statistical Analysis

Clinical and demographic data were extracted from medical records and included: age at diagnosis, tumor histological subtype, presence of metastatic disease at diagnosis, surgical approach, NACT regimen and number of cycles, ACT regimen and number of cycles, and individual drug cumulative doses per BSA.

Baseline characteristics were compared across tumor subtypes using the Kruskal–Wallis test for continuous variables and the Fisher exact test or chi-squared test for categorical variables, as appropriate. Within-patient changes in AMH across serial time points were evaluated using the Wilcoxon signed-rank test for paired samples. Comparisons of AMH levels between tumor subtypes at each time point were performed using the Mann–Whitney U test or Kruskal–Wallis test. The percentage decline in AMH from baseline was calculated for each patient and time point.

Because absolute AMH change is mathematically dependent on baseline AMH, we employed an analysis of covariance (ANCOVA) framework in which post-NACT AMH was modeled as the dependent variable and baseline AMH was entered as a mandatory covariate. Given the right-skewed distribution of AMH, both post-NACT AMH and baseline AMH were natural log-transformed prior to multivariable modeling to better satisfy the normality assumption of linear regression. Candidate predictors included age at diagnosis, pathology type, and cumulative dose per BSA for cyclophosphamide, ifosfamide, platinum agents, and anthracyclines; cyclophosphamide dose was set to zero for patients who did not receive cyclophosphamide during NACT. Osteosarcoma was used as the reference group for pathology comparisons. Multicollinearity among predictors was assessed using the variance inflation factor (VIF), with VIF > 10 considered indicative of severe multicollinearity. The potential interaction between age and pathology type was evaluated by adding product terms to the model and tested via F-test. Because post-NACT AMH values were subject to left-censoring at the assay detection limit of 0.05 ng/mL, we additionally performed a sensitivity analysis using left-censored Tobit regression, with the censoring threshold set at 0.05 ng/mL. Across all serial time points, a total of 148 (16.2% of 912 total measurements) AMH measurements fell below the assay detection limit and were assigned the value of 0.05 ng/mL; of these, 4 occurred at the post-NACT timepoint and were treated as left-censored in the Tobit sensitivity analysis. All statistical analyses were performed using R software (version 4.4.1). All statistical tests were two-sided, and *p* < 0.05 was considered statistically significant.

## 3. Results

### 3.1. Baseline Patient Characteristics and Treatment Exposure

The cohort included 85 female patients with a median age of 12.0 years (range: 4.0–49.0 years). Pediatric patients (≤14 years) comprised 67.1% (*n* = 57) of the cohort, while adolescents (14–18 years) and adults (>18 years) accounted for 12.9% (*n* = 11) and 20.0% (*n* = 17), respectively (Table 1). Osteosarcoma was the predominant diagnosis (76.5%, *n* = 65), with Ewing sarcoma and other sarcomas each representing 11.8% (*n* = 10) of the cohort. Metastatic disease at diagnosis was present in 5.9% (*n* = 5) of the total cohort (Table 1). Surgical intervention was performed in 91.5% (*n* = 75) of patients. Limb-sparing surgery with reconstruction was the most common approach (74.4%, *n* = 61), while limb-sparing surgery without reconstruction was performed in 17.1% (*n* = 14) of cases.

NACT was administered to 92.9% (*n* = 79) of patients. The majority received 3–4 cycles (80.8%, *n* = 63), while 12.8% (*n* = 10) received ≥5 cycles, and 6.4% (*n* = 5) received 1–2 cycles. NACT was administered to 96.9% of osteosarcoma patients (87.3% received 3–4 cycles), 100.0% of Ewing sarcoma patients (67.7% received ≥5 cycles), and 60.0% of other sarcoma patients (Table 1). The median time from baseline to post-NACT cycle 1 assessment was 22 days for osteosarcoma, 19 days for Ewing sarcoma, and 19 days for other sarcomas (Table 2). ACT was received by 92.9% (*n* = 79) of patients, with 67.1% (*n* = 53) receiving >10 cycles and 32.9% (*n* = 26) receiving ≤10 cycles. Among those receiving ACT, the proportion receiving >10 cycles was 71.4% in osteosarcoma patients, 50.0% in Ewing sarcoma patients, and 50.0% in other sarcoma patients (Table 1).

All patients (100.0%, *n* = 85) received anthracyclines and ifosfamide as part of their treatment regimen. Methotrexate was administered to 78.8% (*n* = 67) of patients, with use in 100.0% of osteosarcoma cases and 20.0% of Ewing sarcoma cases. Platinum agents were similarly used in 78.8% (*n* = 67) of patients, administered to all osteosarcoma patients and 20.0% of Ewing sarcoma patients. Cyclophosphamide was used in 21.8% (*n* = 19) of the cohort, including all Ewing sarcoma patients (100.0%) and 54.5% of other sarcoma patients, while being rare in osteosarcoma cases (4.5%). Etoposide was administered to 32.9% (*n* = 28) of patients, with use in all Ewing sarcoma patients (100.0%), 60.0% of other sarcoma patients, and 18.5% of osteosarcoma patients. Vinca alkaloids were used in 72.9% (*n* = 62) of the total cohort (Table 1).

The Alkylating Agent Dose (AAD) score, a cumulative measure of gonadotoxic exposure, was ≥3 in 70.7% (*n* = 58) of patients overall. AAD scores ≥3 were observed in 69.8% of osteosarcoma patients, 100.0% of Ewing sarcoma patients, and 50.0% of other sarcoma patients (Table 1).

**Figure 1:**
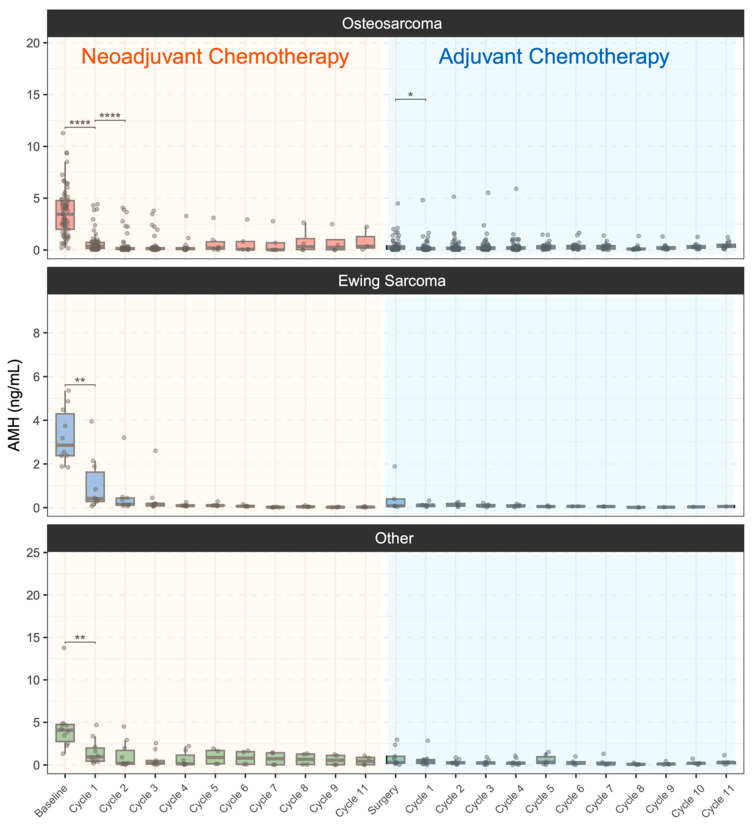
Step-wise AMH Depletion Across Treatment Time Point in Bone and Soft Tissue Sarcoma. Serum AMH levels at baseline and each subsequent treatment time point are shown for the overall cohort and by pathology type. Boxplots represent median and interquartile range; dots represent individual patients. Adjacent time-point comparisons were performed using the Wilcoxon signed-rank test; asterisks indicate statistical significance (* *p* < 0.05, ** *p* < 0.01, **** *p* < 0.001). Time points: Baseline (BL), neoadjuvant chemotherapy cycles 1–9 and 11 (NACT cycle 1–11), surgery (Surg), adjuvant chemotherapy cycles 1–11 (ACT cycle 1–11).

The median baseline AMH level for the entire cohort was 3.5 ng/mL (range: 0.2–13.8 ng/mL) (Table 1, Figure 1). This value is consistent with normative AMH levels reported for age-matched healthy females in the same age range, indicating that ovarian reserve was not systematically compromised prior to treatment initiation in this cohort [14,15,16]. Baseline AMH values were 3.5 ng/mL (range: 0.2–11.3 ng/mL) in osteosarcoma patients, 2.9 ng/mL (range: 1.9–5.4 ng/mL) in Ewing sarcoma patients, and 4.1 ng/mL (range: 1.3–13.8 ng/mL) in other sarcoma patients.

### 3.2. Dynamic Changes in Ovarian Reserve Function

#### 3.2.1. Acute Decline During Neoadjuvant Chemotherapy

AMH levels showed a marked and statistically significant decline following the initiation of NACT. In the overall cohort, median AMH decreased from a baseline of 3.46 ng/mL to 0.39 ng/mL after NACT cycle 1 (88.7% reduction) and further to 0.15 ng/mL after NACT cycle 2 (95.7% reduction from baseline) (Table 2).

**Figure 2:**
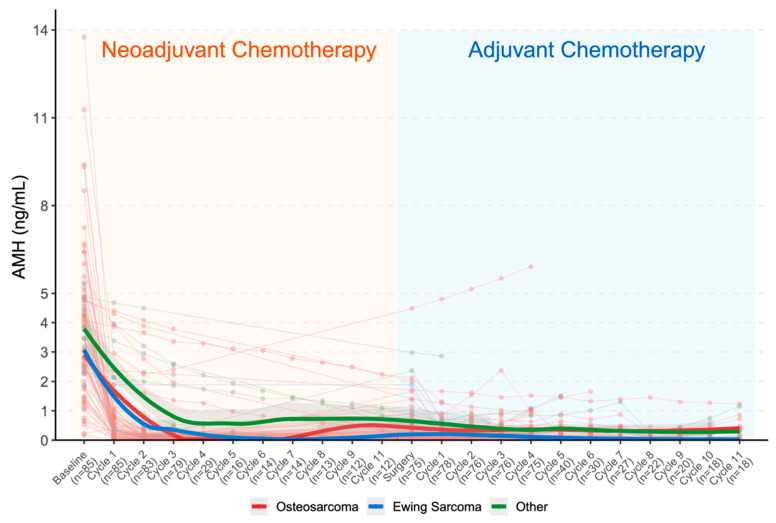
Individual and Mean AMH Trajectories During Neoadjuvant and Adjuvant Chemotherapy by Pathology Type. Individual (thin lines) and loess-smoothed mean (thick lines with shaded area representing 95% CI) AMH trajectories from baseline through adjuvant chemotherapy cycle 11 are shown for Osteosarcoma, Ewing Sarcoma, and Other sarcoma patients. The orange shaded region indicates the neoadjuvant chemotherapy phase; the blue shaded region indicates the adjuvant chemotherapy phase. The vertical dashed line marks surgery. Sample sizes (*n*) at each time point are annotated below the x-axis. AMH suppression was near-maximal within the first two NACT cycles across all pathology subtypes.

This pattern was observed across all tumor subtypes (Figure 1). The longitudinal trajectory of AMH across the neoadjuvant and adjuvant treatment course, together with the number of evaluable samples at each time point, is shown in Figure 2. In the osteosarcoma patients, baseline AMH of 3.45 ng/mL (IQR: 2.00–4.77) declined to 0.35 ng/mL (IQR: 0.15–0.73) after NACT cycle 1, representing an 89.9% reduction (*p* < 0.001). And, in the Ewing sarcoma cohort, baseline AMH of 2.86 ng/mL (IQR: 2.38–4.30) decreased to 0.41 ng/mL (IQR: 0.28–1.62) after NACT cycle 1, representing an 85.7% reduction (*p* < 0.001). In other sarcomas cohort, baseline AMH of 4.08 ng/mL (IQR: 2.75–4.75) declined to 0.96 ng/mL (IQR: 0.44–2.00) after NACT cycle 1, representing an 76.4% reduction (*p* < 0.001).

#### 3.2.2. Risk Factors for Ovarian Reserve Impairment During Neoadjuvant Chemotherapy

**Figure 3:**
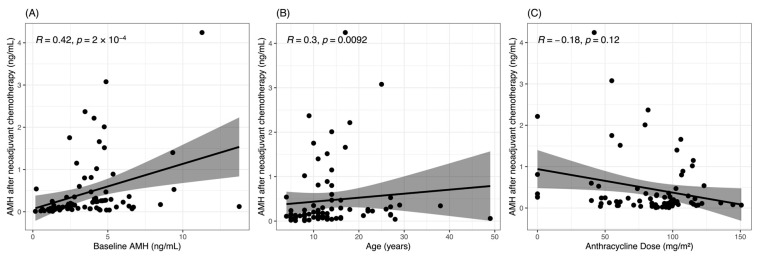
Correlations Between Baseline Parameters and AMH After Neoadjuvant Chemotherapy. Spearman correlation analysis between (**A**) baseline AMH, (**B**) age, and (**C**) anthracycline dose versus AMH after neoadjuvant chemotherapy. Complete univariable Spearman correlation results for others candidate variables, including drug doses, are summarized in Supplementary Figure S2. In the summary table, “Value” refers to the Spearman correlation coefficient (R), and the corresponding two-sided *p*-value is reported alongside it. The solid black line indicates the linear regression line, and the shaded area represents the 95% confidence interval of the regression line.

Among 75 patients with complete paired baseline and post-neoadjuvant chemotherapy (post-NACT) AMH measurements, serum AMH declined substantially during neoadjuvant chemotherapy. In this paired cohort, post-NACT AMH was defined as the AMH measurement obtained at the surgery/post-neoadjuvant time point after completion of neoadjuvant chemotherapy. Median baseline AMH was 3.81 ng/mL, which declined to 0.19 ng/mL post NACT, corresponding to a median absolute decline of 2.75 ng/mL (IQR, 1.80–4.40) and a median percent decline of 94.4% (Table 3). These findings indicate that the marked suppression of ovarian reserve observed during early neoadjuvant treatment persisted through completion of neoadjuvant chemotherapy in the paired analytic cohort. In univariable correlation analysis, baseline AMH was positively associated with post-NACT AMH (Figure 3A), while age was also significantly associated with post-NACT AMH (Figure 3B). Anthracycline dose showed a weak negative correlation with post-NACT AMH, but this association did not reach statistical significance (R = −0.18, *p* = 0.12) (Figure 3C). Similarly, platinum dose showed a comparably weak negative correlation with post-NACT AMH (R = −0.19, *p* = 0.10; Supplementary Figure S2C), with no statistically significant association observed for any individual chemotherapy agent in univariable analysis.

**Figure 4:**
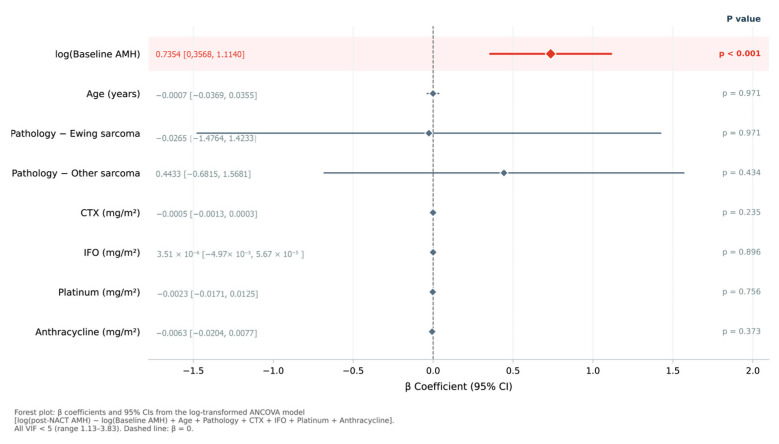
Multivariable ANCOVA Predictors of AMH After Neoadjuvant Chemotherapy. Forest plot showing β coefficients and 95% confidence intervals for each variable in the full ANCOVA model (log(post-NACT AMH) ~ log(baseline AMH) + age + pathology type + anthracycline dose + cyclophosphamide dose + ifosfamide dose + platinum dose).

Because absolute AMH change is mathematically dependent on baseline AMH, post-NACT AMH was selected as the primary outcome for multivariable modeling. In the log-transformed multivariable linear model, log(baseline AMH) was the sole variable independently associated with log(post-NACT AMH) (β = 0.735, 95% CI 0.357–1.114, *p* < 0.001). No treatment-related covariate—including cyclophosphamide dose, ifosfamide dose, platinum dose, or anthracycline dose—reached statistical significance in the adjusted model. Age and pathology type were similarly non-significant. All VIF values were below 5 (range 1.13–3.83), indicating no meaningful multicollinearity among predictors. No significant interaction between age and pathology type was detected (F(2,64) = 0.968, *p* = 0.385). The log-transformed model explained 30.0% of variance in post-NACT AMH (Adj R^2^ = 0.215), compared with 20.3% in the untransformed model (Adj R^2^ = 0.107; Supplementary Table S1), with substantially improved residual normality (Shapiro–Wilk W = 0.961, *p* = 0.020 vs. W = 0.823, *p* < 0.001). The estimates of the primary log-transformed multivariable linear model are summarized in Figure 4 and Table 4.

**Figure 5:**
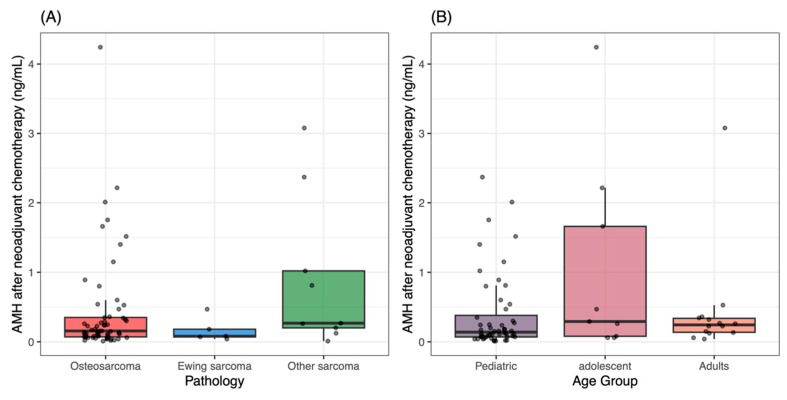
Post-NACT AMH according to pathology type and age group. (**A**) Distribution of post-NACT AMH across pathology groups (osteosarcoma, Ewing sarcoma, and other sarcoma subtypes). (**B**) Distribution of post-NACT AMH across age groups (pediatric, adolescent, and adult). Post-NACT AMH was defined as the AMH measurement obtained at the surgery/post-neoadjuvant time point after completion of neoadjuvant chemotherapy.

To further evaluate the robustness of the multivariable findings, we performed a sensitivity analysis using left-censored Tobit regression with the censoring threshold set at the assay detection limit. In the final paired cohort, 4 post-NACT AMH values were treated as left-censored at 0.05 ng/mL. The overall pattern of associations remained consistent with that of the primary linear model. Baseline AMH remained significantly associated with post-NACT AMH in the Tobit model (estimate = 0.100, 95% CI 0.044 to 0.155; *p* < 0.001). Cyclophosphamide dose, age, pathology type, ifosfamide dose, platinum dose, and anthracycline dose remained non-significant, although the comparison between Ewing sarcoma and osteosarcoma showed a borderline trend (*p* = 0.097). The results of the Tobit sensitivity analysis are provided in Supplementary Table S1. A forest plot of the Tobit model is provided in Supplementary Figure S3. Post-NACT AMH distributions according to pathology type and age group are shown in Figure 5.

Taken together, these analyses support two main points. First, baseline ovarian reserve, as reflected by baseline AMH, remained a stable and clinically meaningful predictor of ovarian reserve at the completion of neoadjuvant chemotherapy. Second, no treatment-related covariate was independently associated with post-NACT AMH in the primary log-transformed model or the Tobit sensitivity analysis, consistently supporting baseline ovarian reserve as the sole robust determinant of residual ovarian reserve after neoadjuvant chemotherapy.

## 4. Discussion

This is the first sarcoma-specific longitudinal AMH study with serial intra-NACT measurements in bone and soft tissue sarcoma. Our central finding is that the vast majority of ovarian reserve depletion occurs within the first one to two NACT cycles—a period we term the acute injury phase. Median AMH declined from 3.46 ng/mL at baseline to 0.39 ng/mL after NACT cycle 1 (88.7% reduction) and 0.15 ng/mL after NACT cycle 2 (95.7% reduction). This ultra-early, near-total suppression challenges the conventional assumption that gonadotoxic damage accumulates gradually across the full treatment course. These findings have important clinical implications. Fertility preservation counseling should be initiated as early as possible after diagnosis, and time-sensitive fertility preservation interventions should ideally be considered before the first NACT cycle whenever clinically feasible. This pattern is consistent with the acute AMH decline observed in breast cancer cohorts by Dezellus et al. [7] and lymphoma cohorts by Decanter et al. [8], but is demonstrated here for the first time in sarcoma with intra-NACT temporal resolution.

In the current multivariable analyses, baseline AMH remained the most robust predictor of post-NACT AMH. This finding is biologically plausible and consistent with the broader oncofertility literature, as baseline ovarian reserve strongly constrains residual ovarian reserve after chemotherapy exposure. In the present paired cohort, no treatment-related covariate reached independent statistical significance in the log-transformed multivariable model. Cyclophosphamide dose, which appeared nominally significant in the untransformed analysis (Supplementary Table S1), was not replicated in the log-transformed model (β = −0.001, *p* = 0.235), suggesting that the original signal reflected the combined influence of AMH non-normality, regimen-structured collinearity, and the small number of cyclophosphamide-treated patients (*n* = 12/75) rather than a true independent pharmacological effect.

The lack of significant associations for platinum and anthracycline doses with post-NACT AMH is mechanistically consistent with published evidence. Anthracyclines exert cytotoxicity primarily through topoisomerase II inhibition in actively dividing granulosa cells of growing follicles, with comparatively limited direct damage to the mitotically quiescent primordial follicle pool reflected by circulating AMH [17,18]. For platinum agents, the absence of a detectable dose–response signal likely reflects regimen collinearity—cisplatin was administered exclusively within the MAP backbone in osteosarcoma patients—rather than true gonadotoxic inactivity. A floor effect arising from near-universal AMH suppression at the post-NACT timepoint further limits the discriminatory capacity of dose–response analyses for any individual agent.

To further examine the stability of these findings, we performed a left-censored Tobit sensitivity analysis using the assay detection limit as the censoring threshold. In the final paired cohort, 4 post-NACT AMH values were treated as left-censored at 0.05 ng/mL. The overall pattern remained consistent with that of the primary linear model, with baseline AMH remaining significant and all treatment-related covariates remaining non-significant. These findings support the robustness of the main multivariable results in the presence of limited left-censoring and reinforce the central role of baseline ovarian reserve in determining post-treatment ovarian reserve.

The ultra-rapid AMH decline observed in this cohort is biologically instructive. AMH is secreted by preantral and small antral follicles, and serum AMH is a sensitive marker of the growing follicle pool. The conventional view holds that chemotherapy damages the ovarian reserve primarily through direct cytotoxicity to growing follicles and granulosa cells [19]. However, the acute AMH suppression within the first one to two cycles—prior to high cumulative dose accumulation—is difficult to reconcile with this mechanism alone. Preclinical evidence indicates that chemotherapy simultaneously triggers the ‘burnout’ pathway: alkylating agents, particularly cyclophosphamide, activate the PI3K/Akt/mTOR signaling axis within dormant primordial follicles, disrupting their quiescence and causing premature recruitment [20,21,22,23]. These mechanisms may accelerate depletion of the dormant follicle pool and thereby contribute to the profound early decline in circulating AMH observed during treatment. Cisplatin exerts gonadotoxic effects through overlapping pathways, including direct DNA double-strand breaks in primordial follicle oocytes, activation of the CHK2-p53/TAp63alpha apoptotic cascade, and PI3K/Akt/FOXO3a-mediated loss of follicular dormancy [23,24,25]. These dual mechanisms—direct oocyte apoptosis and aberrant follicular activation—operate in parallel during chemotherapy exposure [19,23,24,26], jointly accounting for the steep and early AMH decline observed. The convergence between our observational data and these experimental findings supports that gonadotoxic injury is initiated at the very start of treatment, not progressively over its course.

These findings have direct clinical relevance. Nearly all patients experienced severe AMH depletion within two NACT cycles, irrespective of regimen. Fertility preservation counseling should be initiated immediately upon diagnosis, with early intervention strongly considered, as endorsed by the 2025 ASCO guideline update. Fertility preservation strategies should be individualized and stratified according to pubertal status and the urgency of treatment initiation. For prepubertal girls, ovarian tissue cryopreservation is currently the principal fertility preservation option. For post-pubertal patients who can undergo ovarian stimulation without compromising oncologic management, mature oocyte cryopreservation may be considered, with embryo cryopreservation as an option when applicable. For patients receiving highly alkylating agent-intensive regimens, ovarian tissue cryopreservation may also warrant consideration given the severity of early depletion. Serial AMH monitoring during and after treatment may help guide longitudinal reproductive counseling and long-term reproductive planning.

Several strengths merit acknowledgment. This is, to our knowledge, the first sarcoma-specific longitudinal AMH study with serial intra-NACT measurements, providing temporal resolution of ovarian reserve depletion previously uncharacterized in this population. All AMH measurements were performed in a single accredited laboratory using a consistent assay platform, minimizing inter-assay variability. The analytical framework incorporated log-transformation to address AMH’s distributional properties, Tobit sensitivity analysis for left-censored values, VIF assessment for multicollinearity, and interaction testing—collectively providing a robust multivariable analysis despite the real-world, retrospective design.

Several limitations should be acknowledged. This was a single-center study with a predominantly pediatric and adolescent cohort, and generalizability to older adult sarcoma patients remains uncertain. In addition, pubertal status and menarche status were not uniformly available in this retrospective cohort, which may limit interpretation of absolute AMH values across the full age spectrum. Beyond pubertal status, other established determinants of baseline AMH—including BMI, smoking, and hormonal contraceptive use—were not systematically recorded in this retrospective cohort [27,28]. The clinical impact of these factors is nevertheless likely limited, given the predominantly pediatric population and the ethnic homogeneity of this single-center Chinese cohort. Although multivariable modeling was performed, the cohort size remained modest for evaluating regimen-structured drug exposures, and residual confounding cannot be excluded. With 8 predictors and *n* = 75 in the paired cohort, the events-per-variable ratio of 9.4 falls marginally below the recommended threshold of 10, which may modestly limit the precision of individual drug-specific estimates. AMH is a surrogate marker of ovarian reserve and does not directly measure oocyte quality, pregnancy potential, or live-birth outcomes. Follow-up duration was also insufficient to determine whether partial AMH recovery in some patients represents durable recovery or only transient fluctuation.

Future research should determine whether the nadir AMH value or the recovery trajectory within the first 3–6 months predicts long-term fertility outcomes, including spontaneous pregnancy and live birth rates. Multi-center studies with standardized regimens are needed to validate drug-specific gonadotoxic effects in sarcoma. Prospective investigation of biomarker-guided follow-up and early intervention strategies may ultimately improve reproductive outcomes for adolescent and young adult cancer survivors.

## 5. Conclusions

In female patients with bone and soft tissue sarcoma, ovarian reserve depletion occurred predominantly within the first one to two cycles of neoadjuvant chemotherapy. In the paired cohort, AMH remained markedly suppressed at the completion of neoadjuvant treatment. Baseline AMH was the sole independent predictor of post-NACT ovarian reserve in the log-transformed multivariable analysis, with no treatment-related covariate reaching statistical significance in the fully adjusted model. These findings support early fertility preservation counseling and individualized fertility preservation strategies according to pubertal status and treatment urgency. Future multi-center prospective studies are needed to validate drug-specific gonadotoxic effects in sarcoma and to determine whether nadir AMH predicts long-term fertility outcomes in this population.

## Figures and Tables

**Table 1:** Baseline clinicopathologic characteristics of the cohort.

Characteristics	Overall	Osteosarcoma	Ewing Sarcoma	Other Sarcomas	*p* ^2^
	*n* = 85 ^1^	*n* = 65 ^1^	*n* = 10 ^1^	*n* = 10 ^1^	
**Age (years)**					
Median (Range)	12.0 (4.0, 49.0)	13.0 (4.0, 49.0)	12.0 (10.0, 37.0)	16.0 (8.0, 35.0)	0.60
Pediatric (≤14 y)	57 (67.1)	45 (69.2)	7 (70.0)	5 (50.0)	0.13
Adolescent (14–18 y)	11 (12.9)	9 (13.8)	2 (20.0)	0	
Adults (>18 y)	17 (20.0)	11 (16.9)	1 (10.0)	5 (50.0)	
**Baseline AMH (ng/mL)**					
Median (Range)	3.5 (0.2, 13.8)	3.5 (0.2, 11.3)	2.9 (1.9, 5.4)	4.1 (1.3, 13.8)	0.70
**Tumor Site**					
Femur	39 (45.9)	35 (53.8)	2 (20.0)	2 (20.0)	<0.001
Tibia/Fibula	16 (18.8)	13 (20.0)	1 (10.0)	2 (20.0)	
Humerus	13 (15.3)	12 (18.5)	0	1 (10.0)	
Axial Skeleton	13 (15.3)	5 (7.7)	7 (70.0)	1 (10.0)	
Soft Tissue	4 (4.7)	0	0	4 (40.0)	
**Metastasis at Diagnosis**					
Yes	5 (5.9)	2 (3.1)	2 (20.0)	1 (10.0)	0.08
No	80 (94.1)	63 (96.9)	8 (80.0)	9 (90.0)	
**Surgery**					
Limb-sparing surgery with reconstruction	61 (74.4)	55 (87.3)	2 (20.0)	4 (44.4)	<0.001
Limb-sparing surgery without reconstruction	14 (17.1)	6 (9.5)	3 (30.0)	5 (55.6)	
No surgery	7 (8.5)	2 (3.2)	5 (50.0)	0	
Unknown	3	2	0	1	
**Received NACT**					
Yes	79 (92.9)	63 (96.9)	10 (100.0)	6 (60.0)	0.004
1–2 cycles	5 (6.4)	4 (6.3)	0	1 (16.7)	
3–4 cycles	63 (80.8)	55 (87.3)	3 (33.3)	5 (83.3)	<0.001
≥5 cycles	10 (12.8)	4 (6.3)	6 (67.7)	0	
Unknown	1	0	1	0	
No	6 (7.1)	2 (3.1)	0	4 (40.0)	
**Received ACT**					
Yes	79 (92.9)	63 (96.9)	6 (60.0)	10 (100.0)	0.004
≤10 cycles	26 (32.9)	18 (28.6)	3 (50.0)	5 (50.0)	
>10 cycles	53 (67.1)	45 (71.4)	3 (50.0)	5 (50.0)	0.004
No	6 (7.1)	2 (3.1)	4 (40.0)	0	
**AAD Score**					
<3	24 (29.3)	19 (30.2)	0	5 (50.0)	0.044
≥3	58 (70.7)	44 (69.8)	9 (100.0)	5 (50.0)	
Unknown	3	2	1	0	
**MTX**					
Yes	67 (78.8)	65 (100.0)	2 (20.0)	0	<0.001
No	18 (21.2)	0	8 (80.0)	10 (100.0)	
**Anthracyclines**					
Yes	85 (100.0)	65 (100.0)	10 (100.0)	10 (100.0)	>0.99
**Platinum Agents**					
Yes	67 (78.8)	65 (100.0)	2 (20.0)	0	<0.001
No	18 (21.2)	0	8 (80.0)	10 (100.0)	
**Vinca Alkaloids**					
Yes	62 (72.9)	46 (70.8)	10 (100.0)	6 (60.0)	0.08
No	23 (27.1)	19 (29.2)	0	4 (40.0)	
**CTX**					
Yes	19 (21.8)	3 (4.5)	10 (100.0)	6 (54.5)	<0.001
No	68 (78.2)	63 (95.5)	0	5 (45.5)	
**IFO**					
Yes	85 (100.0)	65 (100.0)	10 (100.0)	10 (100.0)	>0.99
**VP16**					
Yes	28 (32.9)	12 (18.5)	10 (100.0)	6 (60.0)	<0.001
No	57 (67.1)	53 (81.5)	0	4 (40.0)	

^1^ Median (Min, Max); *n* (%); ^2^ Kruskal–Wallis rank sum test; Fisher’s exact test; AMH: anti-Müllerian hormone; NACT: neoadjuvant chemotherapy; ACT: adjuvant chemotherapy; AAD: alkylating agent dose; MTX: Methotrexate; CTX: Cyclophosphamide; IFO: Ifosfamide; VP16: Etoposide.

**Table 2:** Evolution of serum AMH level during chemotherapy.

AMH (ng/mL)	Total			Osteosarcoma			Ewing Sarcoma			Other Sarcoma		
	No. of Patients	Days * (Median)	AMH (Median (IQR))	No. of Patients	Days * (Median)	AMH (Median (IQR))	No. of Patients	Days * (Median)	AMH (Median (IQR))	No. of Patients	Days * (Median)	AMH (Median (IQR))
Baseline	85	0	3.46 (2.28–4.77)	65	0	3.45 (2.00–4.77)	10	0	2.86 (2.38–4.30)	10	0	4.07 (2.75–4.75)
Post-NACT cycle 1	85	21	0.39 (0.19–0.83)	65	21	0.35 (0.15–0.73)	10	20	0.41 (0.28–1.62)	10	20	0.96 (0.44–2.00)
Post-NACT cycle 2	83	45	0.15 (0.08–0.28)	64	45	0.13 (0.07–0.21)	9	38	0.15 (0.12–0.44)	10	43	0.20 (0.13–1.72)
Surgery (Post-NACT)	75	70	0.19 (0.08–0.51)	61	70	0.17 (0.08–0.39)	5	70	0.08 (0.07–0.40)	9	70	0.29 (0.21–1.02)
Post-ACT cycle 2	76	108	0.14 (0.08–0.26)	62	108	0.14 (0.08–0.26)	5	108	0.13 (0.07–0.19)	9	108	0.24 (0.16–0.37)
Post-ACT cycle 4	75	150	0.16 (0.08–0.29)	61	150	0.16 (0.08–0.30)	5	150	0.07 (0.06–0.12)	9	150	0.23 (0.11–0.28)
Post-ACT cycle 6	30	189	0.21 (0.09–0.42)	23	195	0.24 (0.11–0.43)	2	224	0.06 (0.06–0.06)	5	188	0.22 (0.09–0.38)
Post-ACT cycle 8	22	228	0.08 (0.06–0.14)	15	235	0.10 (0.07–0.17)	2	289	0.05 (0.05–0.05)	5	227	0.07 (0.07–0.13)
Post-ACT cycle 10	18	272	0.22 (0.10–0.37)	12	276	0.26 (0.17–0.41)	1	299	0.05 (0.05–0.05)	5	272	0.20 (0.10–0.21)

AMH, anti-Müllerian hormone; IQR, interquartile range; NACT: neoadjuvant chemotherapy; ACT: adjuvant chemotherapy. * Days: median days from first chemotherapy. Values below the assay detection limit were displayed as 0.05 ng/mL.

**Table 3:** Characteristics of the Paired AMH Cohort (*n* = 75).

Characteristics	No. (%) or Median (IQR)
**Pathology, *n* (%)**	
Osteosarcoma	61 (81.3)
Ewing sarcoma	5 (6.7)
Other sarcoma	9 (12.0)
**Age (years), median (IQR)**	12.0 (8.5–16.0)
**Age group, *n* (%)**	
Pediatric	52 (69.3)
Adolescent	9 (12.0)
Adults	14 (18.7)
**AMH levels**	
Baseline AMH (ng/mL), median (IQR)	3.81 (2.30–4.78)
Post-NACT AMH (ng/mL), median (IQR)	0.19 (0.07–0.41)
AMH change (ng/mL), median (IQR)	−2.8 (1.8–4.4)
AMH decline rate (%), median (IQR)	94.4 (87.6–97.1)
Paired Wilcoxon *p*	<0.001
**NACT cumulative doses per BSA**	
CTX (mg/m^2^), median (IQR)	1174.9 (1038.2–2018.0) †
IFO (mg/m^2^), median (IQR)	11,855.4 (10,588.3–14,803.6)
Platinum (mg/m^2^), median (IQR)	66.8 (35.9–78.5)
Anthracycline (mg/m^2^), median (IQR)	88.8 (60.5–102.7)

AMH, anti-Müllerian hormone; IQR, interquartile range; NACT: neoadjuvant chemotherapy; BSA: body surface area; CTX: Cyclophosphamide; IFO: Ifosfamide. † Only patients received CTX during neoadjuvant chemotherapy period were included to calculate CTX cumulative doses per BSA (*n* = 12); Post-NACT: surgery/post-neoadjuvant time point in the paired cohort. Values are presented as median (IQR) for continuous variables and *n* (%) for categorical variables. AMH change represents absolute decline from baseline to post-NACT timepoint.

**Table 4:** Factors Associated with Post-NACT AMH (Log-transformed Multivariable Model, *n* = 75).

Variable	β	95% CI	*p*
Baseline AMH (ng/mL)	0.7354	0.3568 to 1.1140	<0.001
Age (years)	−0.0007	−0.0369 to 0.0355	0.97
Pathology (ref: Osteosarcoma)			
Ewing sarcoma	−0.0265	−1.4764 to 1.4233	0.97
Other sarcoma	0.4433	−0.6815 to 1.5681	0.43
CTX (mg/m^2^)	−0.0005	−0.0013 to 0.0003	0.24
IFO (mg/m^2^)	3.51 × 10^−6^	−4.97 × 10^−5^ to 5.67 × 10^−5^	0.90
Platinum (mg/m^2^)	−0.0023	−0.0171 to 0.0125	0.76
Anthracycline (mg/m^2^)	−0.0063	−0.0204 to 0.0077	0.37

AMH, anti-Müllerian hormone; NACT: neoadjuvant chemotherapy; CTX: Cyclophosphamide; IFO: Ifosfamide; β: regression coefficient; CI: confidence interval. Outcome variable: natural log-transformed post-NACT AMH. Baseline AMH was similarly log-transformed. R^2^ = 0.300, Adj R^2^ = 0.215. All VIF < 5 (range 1.13–3.83). CTX dose was set to 0 for patients not receiving cyclophosphamide during NACT.

## Data Availability

The data presented in this study are available on reasonable request from the corresponding authors. The data are not publicly available due to privacy and ethical restrictions.

## Supplementary Materials

The following supporting information can be downloaded at: https://www.mdpi.com/article/10.3390/cancers18111821/s1, Figure S1: CONSORT-style flow diagram showing patient screening, exclusion, final inclusion, and derivation of the analysis cohorts. A total of 92 female patients with histologically confirmed bone or soft tissue sarcoma who underwent serum AMH testing were screened. After exclusion of 7 patients, 85 were included in the longitudinal cohort, 79 were included in the serial AMH trajectory analyses, and 75 formed the paired cohort for risk factor analyses of post-NACT AMH; Figure S2: Correlations Between Drug Doses and AMH After Neoadjuvant Chemotherapy. Spearman correlation analysis between (A) cyclophosphamide dose, (B) ifosfamide dose, and (C) platinum dose versus AMH after neoadjuvant chemotherapy; Figure S3: Forest plot of the left-censored Tobit regression model for post-NACT AMH. The model was fitted using 0.05 ng/mL as the censoring threshold. In the final paired cohort, 4 post-NACT AMH values were treated as left-censored at the assay detection limit. Osteosarcoma was used as the reference group for pathology; Table S1: Sensitivity analysis using left-censored Tobit regression for post-NACT AMH.

## Abbreviations

The following abbreviations are used in this manuscript:

**Abbreviations**	**Full form**
AAD	Alkylating agent dose
ACT	Adjuvant chemotherapy
AMH	Anti-Müllerian hormone
ANCOVA	Analysis of covariance
AYA	Adolescents and young adults
BSA	Body surface area
CI	Confidence interval
CTX	Cyclophosphamide
DDP	Cisplatin
DOX	Doxorubicin
EPV	Events per variable
HD-MTX	High-dose methotrexate
IFO	Ifosfamide
IQR	Interquartile range
MTX	Methotrexate
NACT	Neoadjuvant chemotherapy
POI	Premature ovarian insufficiency
VIF	Variance inflation factor
VP-16	Etoposide
