# Supplementary material for: Early Ovarian Reserve Depletion During Neoadjuvant Chemotherapy in Female Patients with Bone and Soft Tissue Sarcoma: A Longitudinal Anti-Müllerian Hormone Study
Source: Cancers (Basel). 2026 Jun 2;18(11):1821. doi: 10.3390/cancers18111821 (PMC13256773; doi:10.3390/cancers18111821)
Supplement: Supplementary file 1 [file cancers-18-01821-s001.zip › cancers-4313156-supplementary.pdf]

cancers

MDPI

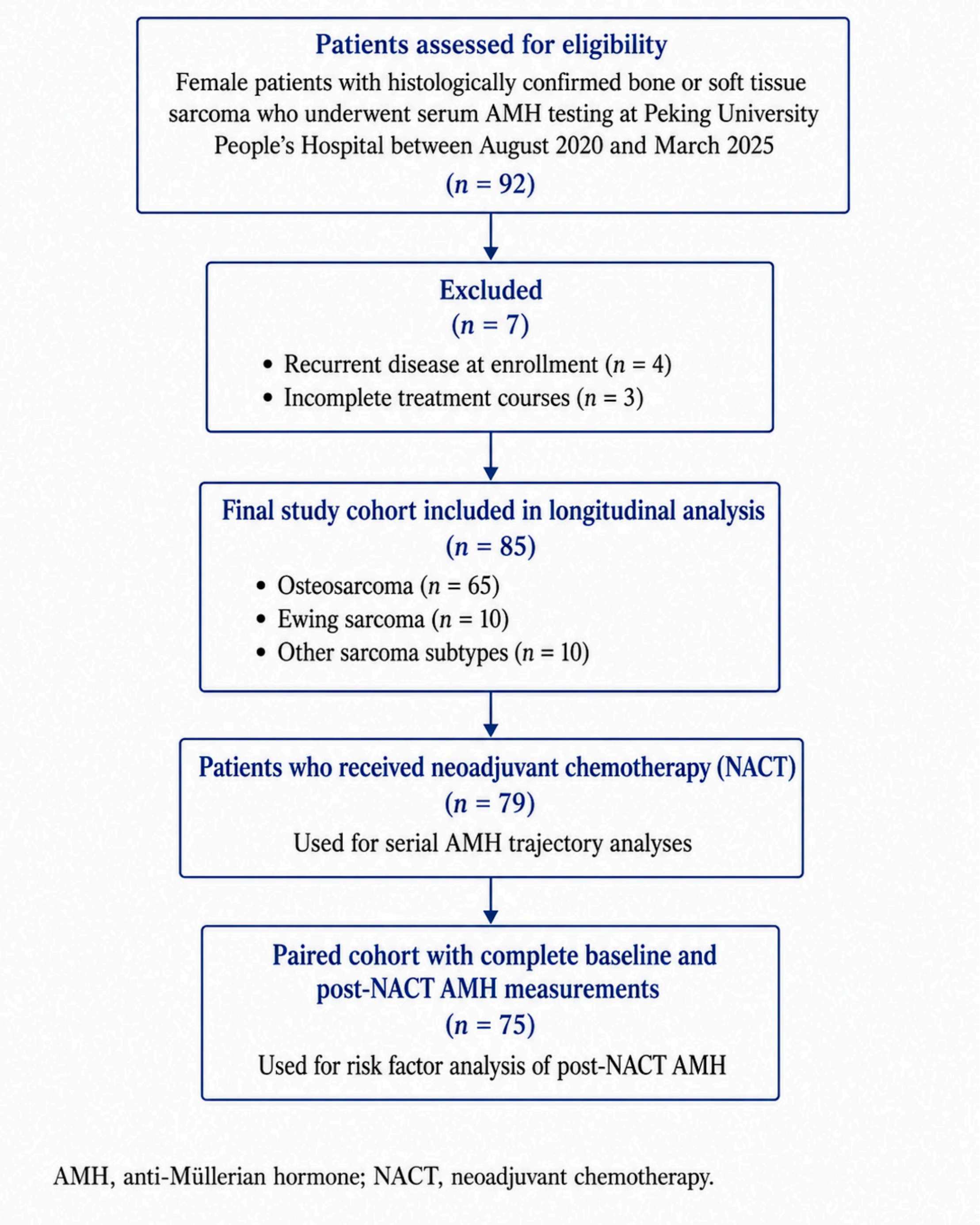

Supplementary Figure S1. CONSORT-style flow diagram showing patient screening, exclusion, final inclusion, and derivation of the analysis cohorts. A total of 92 female patients with histologically confirmed bone or soft tissue sarcoma who underwent serum AMH testing were screened. After exclusion of 7 patients, 85 were included in the longitudinal cohort, 79 were included in the serial AMH trajectory analyses, and 75 formed the paired cohort for risk factor analyses of post-NACT AMH.

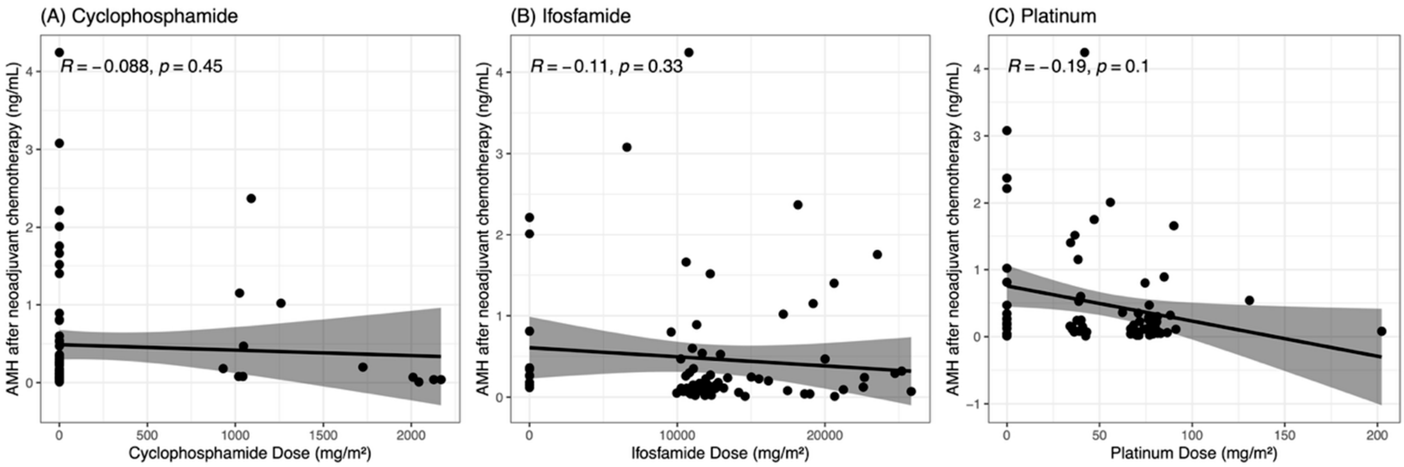

Supplementary Figure S2. Correlations Between Drug Doses and AMH After Neoadjuvant Chemotherapy. Spearman correlation analysis between (A) cyclophosphamide dose, (B) ifosfamide dose, and (C) platinum dose versus AMH after neoadjuvant chemotherapy.

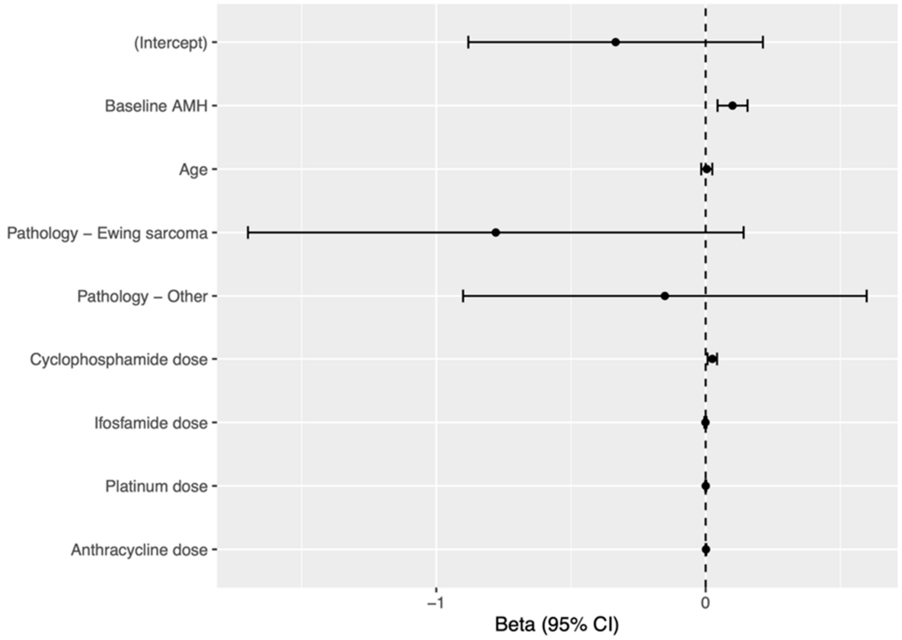

Supplementary Figure S3. Forest plot of the left-censored Tobit regression model for post-NACT AMH. The model was fitted using 0.05 ng/mL as the censoring threshold. In the final paired cohort, 4 post-NACT AMH values were treated as left-censored at the assay detection limit. Osteosarcoma was used as the reference group for pathology.

Supplementary Table S1. Sensitivity analysis using left-censored Tobit regression for post-NACT AMH.

Variable	β	95% CI	P value
Baseline AMH	0.100	0.044 to 0.155	0.00045
Age	0.004	-0.017 to 0.025	0.70287
Pathology – Ewing sarcoma	-0.779	-1.699 to 0.141	0.09697
Pathology – Other	-0.151	-0.901 to 0.598	0.69190
Cyclophosphamide dose	0.025	0.007 to 0.042	0.00578
Ifosfamide dose	-0.00075	-0.00483 to 0.00332	0.71663
Platinum dose	0.00023	-0.00169 to 0.00215	0.81667

https://doi.org/10.3390/cancers18111821

Variable	β	95% CI	P value
Anthracycline dose	0.00115	-0.00043 to 0.00273	0.15241

A left-censored Tobit regression model was fitted using 0.05 ng/mL as the censoring threshold. In the final paired cohort, 4 post-NACT AMH values were treated as left-censored at the assay detection limit. Osteosarcoma was used as the reference group for pathology.

https://doi.org/10.3390/cancers18111821
